# An Examination of Temporomandibular Joint Disc Displacement through Magnetic Resonance Imaging by Integrating Artificial Intelligence: Preliminary Findings

**DOI:** 10.3390/medicina60091396

**Published:** 2024-08-26

**Authors:** Oana Almășan, Sorana Mureșanu, Petra Hedeșiu, Andrei Cotor, Mihaela Băciuț, Raluca Roman, TEAM Project Group

**Affiliations:** 1 Department of Prosthetic Dentistry and Dental Materials, Iuliu Hațieganu University of Medicine and Pharmacy, 400006 Cluj-Napoca, Romania; 2 Department of Maxillofacial Surgery and Radiology, Iuliu Hațieganu University of Medicine and Pharmacy, 37 Iuliu Hossu Street, 400029 Cluj-Napoca, Romania; 3 Emil Racoviță College, 9-11 Mihail Kogălniceanu, 400084 Cluj-Napoca, Romania; 4 Computer Science Department, Babes Bolyai University, 1 Mihail Kogălniceanu, 400084 Cluj-Napoca, Romania

**Keywords:** temporomandibular joint, disc displacement, artificial intelligence, deep learning, magnetic resonance imaging

## Abstract

*Background and Objectives:* This research was aimed at constructing a complete automated temporomandibular joint disc position identification system that could assist with magnetic resonance imaging disc displacement diagnosis on oblique sagittal and oblique coronal images. *Materials and Methods:* The study included fifty subjects with magnetic resonance imaging scans of the temporomandibular joint. Oblique sagittal and coronal sections of the magnetic resonance imaging scans were analyzed. Investigations were performed on the right and left coronal images with a closed mouth, as well as right and left sagittal images with closed and open mouths. Three hundred sagittal and coronal images were employed to train the artificial intelligence algorithm. *Results:* The accuracy ratio of the completely computerized articular disc identification method was 81%. *Conclusions:* An automated and accurate evaluation of temporomandibular joint disc position was developed by using both oblique sagittal and oblique coronal magnetic resonance imaging images.

## 1. Introduction

The term “temporomandibular disorders” encompasses a range of diseases that impact the temporomandibular joint (TMJ) and the tissues related to it [1]. Two global agreement meetings have been conducted to conclude on a novel diagnostic algorithm that would apply to the majority of pain-related temporomandibular disorders (TMDs) [2]. Specialist management for therapy for individuals with discomforting TMDs has been developed [3]. Signs of discomfort and muscle dysfunction can be manifestations of TMDs [4]. Temporomandibular disorders affect 34% of the globe’s demographics, with subjects aged 18 to 60 being the most vulnerable [5]. TMDs constitute a remarkable public health issue; within Confucian heritage cultures, the prevalence of TMDs was 15%, while the frequency of TMD pain constituted 8%, and TMJ sounds reached 24% [6]. Anterior disc displacement is frequently encountered in subjects with TMDs [7]. Research has demonstrated a connection between bruxism, joint conditions, and occlusion characteristics [8]. Determining the disc position of the temporomandibular joint (TMJ) can be evaluated through magnetic resonance imaging (MRI) [9].

Different artificial intelligence (AI) algorithms created for identifying TMDs might offer more scientific knowledge to improve the precision of diagnostics of TMJ-related pathology [10]. There is an increasing focus on research on TMJs and TMDs that makes use of machine learning tools and techniques that employ artificial intelligence in aiding diagnosis [11]. Segmentation algorithms based on convolutional neural networks (CNNs) might serve as a useful instrument to help physicians detect relevant TMJ structures based on magnetic resonance imaging (MRI) scans [12]. Encouraging outcomes using a completely computerized, deep learning (DL)-based approach have been reported [13]. Disk locations on MRI scans might be recognized using a deep learning algorithm based on image segmentation [14]. A completely computerized articular disk recognition method has been suggested for aiding the identification of TMDs in MRI [15].

Scanning investigations, including automated learning techniques, such as support vector machines, k-nearest neighbors, and decision tree algorithms, are being used to identify temporomandibular disorders (TMDs) [16]. Using deep learning algorithms, TMJ pathology can be identified with considerable sensitivity and specificity [17]. Certain models are considered efficient in precise detection [18]. Artificial intelligence (AI)-based technologies can accelerate dental healthcare and enhance accurate and collaborative dentistry [19]. The ability of AI to deliver relevant evidence for TMD detection has been emphasized as well due to model simplicity and cost-effectiveness [16]. To distinguish TMJ’s normal variations and abnormalities into different categories, the DenseNet-121 and InceptionV3 deep learning techniques were employed by B. Thanathornwong et al. [20]. Dong et al. demonstrated that MobileNet-V2 shows promising results for image classification, achieving higher accuracy and shorter training times compared to other models, such as MobileNetVl, Xception [6], Inception-ResNetV2, and ResNetl52 [21].

With the help of transfer learning, pre-trained models such as MobileNet-V2 can be applied to medical image classification tasks as well. This approach also proves valuable in the case of small datasets. These can be handled effectively by transferring knowledge from a related domain where large datasets are available [22].

Machine learning algorithms for the accurate diagnosis of TMD may be improved with additional characteristics in examination scans such as neuroimaging information [23]. The use of MRI and AI for diagnosing forecasts was reported based on a deep learning algorithm for TMJ disc displacement identification [24]. In the diagnosis of TMJ osteoarthritis, AI is anticipated to reduce the subjective nature involved in individual judgment and accelerate the diagnosis [25]. Research on a completely automated, deep learning-based technique using an encoder–decoder convolutional neural network (ED-CNN) model for segmenting TMJ articular discs on MRI delivered encouraging findings [13]. The present research aimed to explore the effectiveness of employing machine learning methods such as MobileNet-V2 with transfer learning to detect the existence of TMJ sagittal or coronal disc displacements in grown-up individuals on a restricted set of data.

## 2. Materials and Methods

The Ethics Committee of the “Iuliu Hațieganu’’ University of Medicine and Pharmacy in Cluj-Napoca, Romania, provided approval to conduct the study (approval number 117/04.06.2024). Each method complied with the 1975 Declaration of Helsinki, as amended in 2008, and the ethical guidelines of the applicable authority on human testing. Each subject was given a research form that included areas for clinical assessments and personal data. The research was observational and cross-sectional.

MRI scans of 50 adult individuals diagnosed with TMD, according to the DC/TMD Axis I criteria [2], were included in the study. Examinations were conducted in a single center in Cluj-Napoca, Romania, between January and March 2024. The images were acquired on a 1.5 T MRI scanner (General Electric, Signa Excite HD, General Electric Healthcare, Helsinki, Finland) with a split head coil. Patients who had undergone orthognathic surgery, were in acute pain, had limited mouth opening, were completely edentulous, or had examinations containing artifacts that prevented accurate assessment were excluded.

Only proton density (PD) sequences were included. Oblique sagittal and coronal sections were exported without compression to BMP files. Following this, the images were cropped to include the condylar head, disc, glenoid fossa, and articular eminence. Observations were performed on bilateral coronal images, as well as on bilateral sagittal images, in an open and closed mouth position, totaling 300 MRI sections or 6 images per patient.

**Figure 1:**
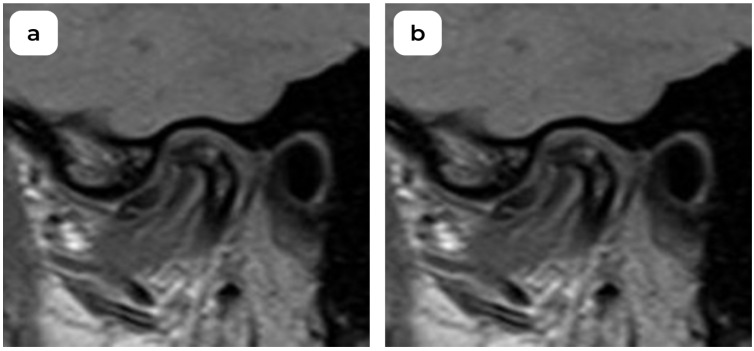
MRI oblique sagittal view of left TMJ: (**a**) anterior disc displacement in a closed-mouth acquisition, (**b**) disc displacement in an open-mouth acquisition (DDWoR).

**Figure 2:**
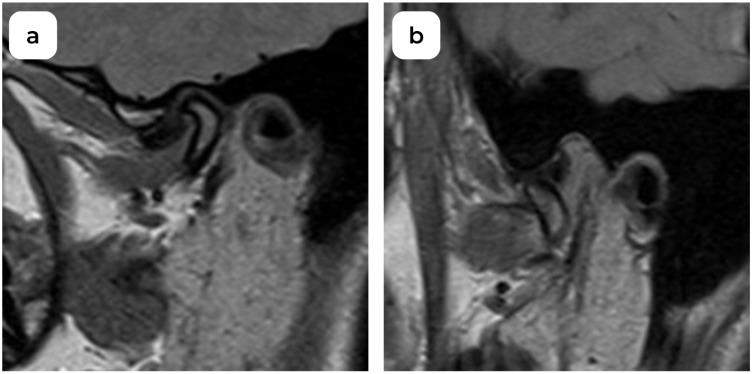
MRI oblique sagittal view left TMJ: (**a**) anterior disc displacement in closed-mouth acquisition; (**b**) open-mouth normal disc acquisition (DDwR).

**Figure 3:**
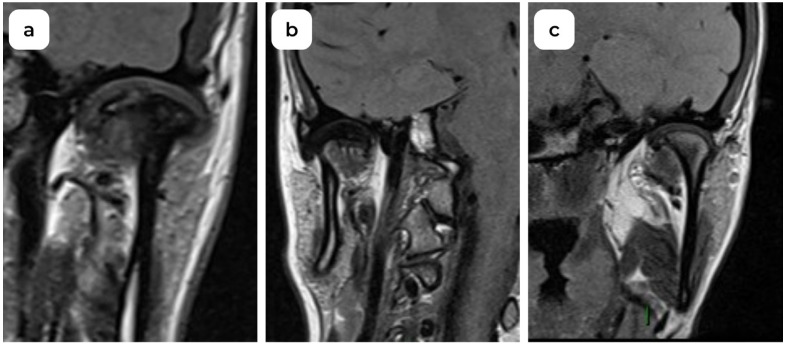
MRI oblique coronal view of TMJ: (**a**) normal disc position; (**b**) lateral disc displacement; (**c**) medial disc displacement.

Labeling was performed by two calibrated researchers: one with over 15 years of experience in reading MRI images (OA) and the other with 5 years of experience (S M) in reading MRI images using the makesense.ai interface [26]. The diagnostic conditions of normal disc position (N), anterior disc displacement with reduction (DDwR), and anterior disc displacement without reduction (DDwoR) were observed in the sagittal sections of the MRI scans (Figure 1 and Figure 2). Posterior disc displacement was not observed. As such, the individual images were labeled as normal (N) or anterior disc displacement (ADD). Normal (N) disc position and medial (MDD) or lateral (LDD) disc displacement were noted in the coronal sections (Figure 3). These annotations served as ground truth. The inter-rater agreement for image assessment was evaluated using SPSS Statistics Version 22.0. Only one label was fed into the artificial intelligence model after the two raters reached an agreement.

**Figure 4:**
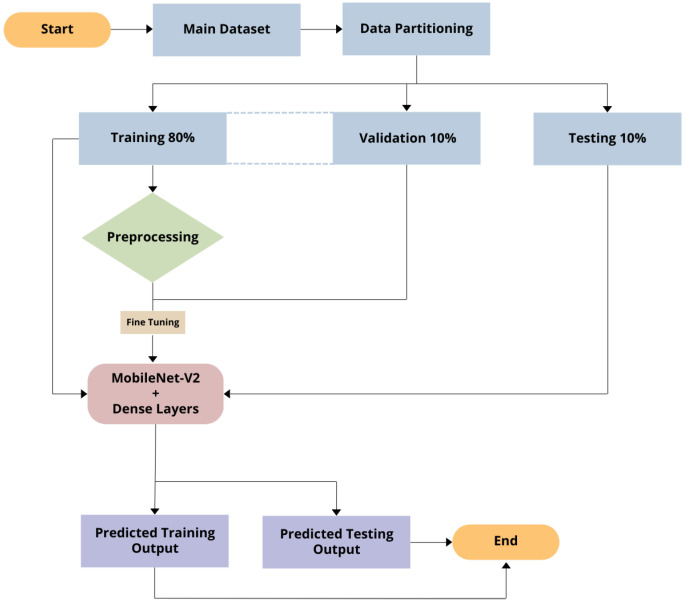
Flowchart of the AI training process. First, the data is split into training, testing, and validation datasets. A MobileNet-V2 model with additional dense layers is applied to the training dataset (Figure 6). The validation dataset is used for fine-tuning during the training process. After training, the predicted outputs are returned by the model.

**Figure 5:**
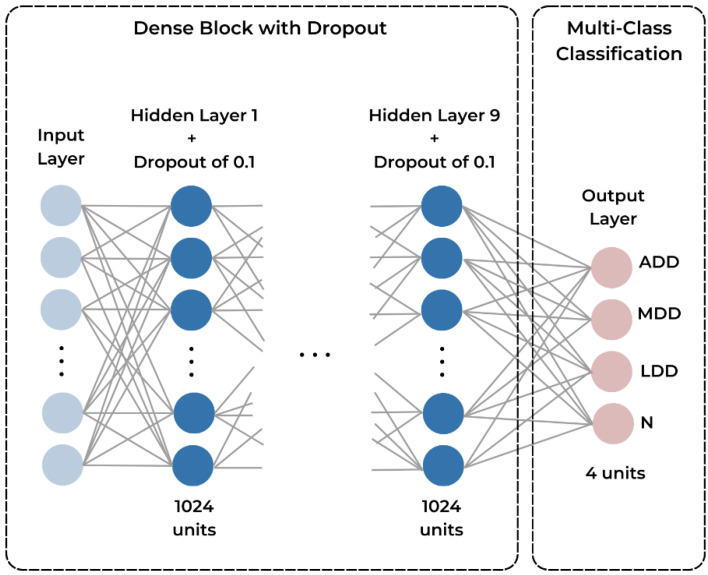
Dense block representation with multi-class classification. Each fully-connected layer is followed by a dropout layer with a rate of 0.1. The output layer has softmax activation and 4 output units for the multi-class classification of the 4 disc displacement categories. ADD—anterior disc displacement, MDD—medial disc displacement, LDD—lateral disc displacement, N—normal.

**Figure 6:**
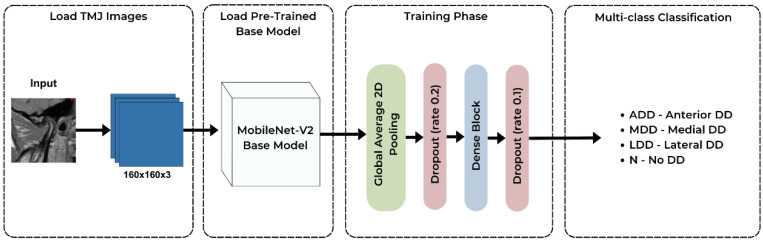
Modified architecture of the MobileNet-V2 model. The input 160 × 160 × 3 image is fed into the pre-trained MobileNet-V2 base model, and transfer-learning is applied. The output has softmax activation and 4 units for multi-class classification. DD—Disc displacement, ADD—anterior disc displacement, MDD—medial disc displacement, LDD—lateral disc displacement, N—normal.

The TMD image classification model was developed in Python using TensorFlow and Keras. The model used transfer learning techniques by employing a modified version of MobileNet-V2 that was pre-trained on ImageNet [27]. A flowchart of the creation of the model can be seen in Figure 4. To prepare the data for training, the 300-image dataset was first shuffled and batched. Each image was resized to 160 × 160 pixels. The data were then split into 80% for training, 10% for testing, and 10% for validation. Prefetching was also employed to optimize data loading times and the training process. Random horizontal flipping and rotation were applied to increase the diversity of the training data. The base model consists of the pre-trained MobileNet-V2 with its top classification layer removed [28]. To this, a global average 2D pooling layer was added to reduce the spatial dimensions of the feature maps, followed by a custom sequential block with dense layers using ReLU activation and a dropout rate of 0.1 for further feature extraction. The final classification layer had four output units (one for each type of disc displacement) and softmax activation, being suitable for multi-class classification tasks, such as TMD classification (Figure 5). The complete model architecture can be seen in Figure 6. Finally, the model was configured with the Adam optimizer, sparse categorical cross-entropy loss, and accuracy as a performance metric. An early stopping callback was also utilized to prevent overfitting in case the validation loss ceased to improve.

## 3. Results

A total of 100 TMJs from 50 patients were analyzed, resulting in a pooled dataset of 300 PD images captured in oblique sagittal and coronal planes. For each patient, six images were evaluated: four in the sagittal plane (with closed and open mouth, for right and left TMJ) and two in the coronal plane (left and right TMJ). Characteristics of the dataset are outlined in Table 1.

The study sample comprised 22% male and 78% female participants, with a mean age of 29.5 years, ranging from 21 to 57. Of the 50 patients, 15 had a unilateral TMJ disc displacement, while 24 had a bilateral TMJ disc displacement. A total of 37 TMJs showed no disc displacement. Disc displacement with reduction was present in 34 TMJs, and displacement with no reduction in 29 TMJs.

Two raters classified a pool of 40 images to check the inter-rater reliability, and a Cohen kappa of 0.9 (*p* < 0.001) was obtained, indicating almost perfect agreement.

**Figure 7:**
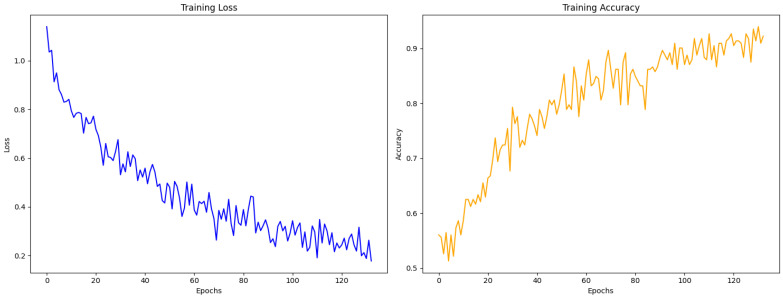
Plots of learning curves for training loss and accuracy.

There was a l6% training loss. To attain this level of precision, 133 epochs were required (with an early stopping at 500 to prevent overfitting). The completely computerized technique for detecting joint disc position had a training accuracy of 92% (Figure 7). Test loss reached 36%, whereas the overall test accuracy for disc displacement classification reached 81%. The learning curves in Figure 7 reveal a steady decrease in training loss and a consistent increase in training accuracy, with no major fluctuations. This, indeed, confirms that the model efficiently improves when diagnosing TMD.

## 4. Discussion

The assessment of disc position in individuals with temporomandibular disorders (TMDs) is of significant clinical importance. Our research presents a viable approach to a comprehensive, computerized evaluation of the temporomandibular joint (TMJ) disc position on MRI images. Notably, our study observed the disc displacement in both coronal and sagittal slices, which constitutes a novel aspect compared to previous studies.

The past few years have seen the ongoing development of several AI algorithms for diagnosing TMD [10,11]. The automatic detection of disk position can assist in the initial detection of TMD and render an easier primary diagnosis [29]. A previous study by Kao et al. introduced an AI-based diagnostic tool for detecting temporomandibular joint disc displacement using structural MRI images [30]. It utilized U-net to identify the area of interest using 100 sagittal MRI images of the TMJ. Their best-performing models, InceptionV3 and DenseNet169, achieved a high accuracy (85%) and precision (86%), demonstrating the potential of automated TMJ disc displacement detection using deep learning. Recently, Kim et al. also used a deep-learning-based approach to predict disc perforations on MRI findings [31]. They achieved superior performance (94% accuracy) compared to conventional methods. Our current model does not detect disc perforations yet, but such an addition could aid in creating a more complete AI-powered medical analysis tool for identifying TMDs.

Lee et al. trained three VGG16-based models to identify the disc displacement of the temporomandibular joint in magnetic resonance imaging on a larger dataset (2520 TMJs), achieving a maximum accuracy of 83%. This model’s key advantage was that it had higher specificity than individual professionals, which may aid in diagnosis [32]. Our model’s accuracy reached a similar performance of 81%, which assists in disc position identification, making it an advantageous resource. Expanding the dataset should improve current results.

Our study employed a modified version of MobileNet-V2, pre-trained on ImageNet, which is designated for general-purpose image classification tasks [21,27]. While Mobilenet-V2 can be adjusted by using transfer learning, its pre-trained weights on ImageNet may not transfer optimally to TMD classification tasks [22]. Moreover, MobileNet-V2 is optimized in terms of efficiency and speed, utilizing depthwise separable convolutions [28]. While this reduces computational power, it may not capture some details necessary for TMD classification, such as joint abnormalities or muscle asymmetries. To address this issue, our team is aiming to develop a custom CNN architecture that would be specifically tailored according to TMD’s characteristics. As such, the model can be designed to focus on relevant anatomical structures.

Similar to the study by Lee et al. [32], we used only proton density (PD)-weighted images (WI) to train the model. PD sequences in MRI provide high-contrast and detailed images of the temporomandibular joint, making them particularly useful for clinical assessments of the articular disc [33]. One study proposed a Pix2Pix generative adversarial network with transfer-learning to synthesize T2-WI based on PD-WI, which proved to be useful for diagnosis [34].

To help medical practitioners evaluate anterior disk displacement before therapy and thereby increase the results of therapy, algorithms that can automatically identify anterior disc displacement of the TMJ on MRIs have been constructed [35]. The quality of life of patients may be improved by the automatic disk position detection, which may help with the clinical assessment of disk position. This could improve diagnosis and expand treatment choices.

AI-driven technology to assist in the initial detection of TMD renders an easier primary diagnosis [29]. The integration of AI-based segmentation tools into diagnostics might ease TMJ examination, especially for the diagnosis of TMD and long-term follow-up [36]. One study applied two convolutional neural networks (CNNs) to semantically segment the mandibular condyle, articular eminence, and TMJ disc in MRI images [12], with the potential to serve as a foundation for additional automated analysis of TMJ pathology.

The study’s limitations include the comparatively limited number of records and the MRI images’ single-center origin, which raises questions regarding the generalizability of the findings. The restricted sample size (300 images) constitutes a primary shortcoming. Small datasets may impose a potential overfitting risk for deep learning models, where the model performs well on training data but poorly on validation or test data. This limitation can be mitigated by utilizing techniques such as data augmentation, dropout, and regularization, but overfitting is still a concern. The better approach is to use more image slices per patient or to include all MRI slices where the disc is visible, rather than just a single slice. Future research should aim to expand the dataset, both in terms of the number of cases and the diversity of their origins, potentially employing data augmentation strategies.

Our team plans to advance this work by adopting a multiparametric approach and incorporating clinical data from medical records. The records will consist of data from the DC/TMD Axis I and II diagnostic algorithms [2]. Medical records have been used in the past for the automated assessment of TMD and orofacial pain [37]. Another potential research avenue is incorporating explainability into the model. In medical AI, understanding a model’s predictions is crucial for clinician trust and adoption, especially amid the growing need to counter the increasing opacity of contemporary models [38]. Techniques like Grad-CAM create heat maps highlighting regions most responsible for the predicted class probability [39]. Yoon et al. applied this technique to TMD diagnosis, providing an explainable clinical decision support system [24].

As such, our algorithm could prove to be valuable for inexperienced clinicians, potentially serving as an educational tool as well. For an efficient integration of the AI model in clinical workflows, more features of the model could be developed. This would provide a complete TMD diagnosis, enhance clinician trust, and ultimately aid in incorporating the model into healthcare institutions.

## 5. Conclusions

The present CNN architecture managed to obtain a high accuracy of 81%, even on a small training dataset. Further studies ought to enlarge the set of images, integrate clinical information, and create a specialized CNN architecture for TMD. Increasing the AI model’s clarity may improve practitioner confidence. The present research shows how AI may be used to enhance TMD diagnosis from MRI images.

## Figures and Tables

**Table 1:** Characteristics of the dataset.

Dataset Characteristics					
MRI Section	Condition		Patients	Condition	TMJs
Sagittal	No DD		11	No DD	37
	ADD	bilateral	24	DDwR	34
		unilateral	15	DDwoR	29
Coronal	No DD		11	No DD	54
	MDD *		20	MDD	25
	LDD *		21	LDD	21
	Total		50	Total	100

DD—Disc displacement, ADD—anterior disc displacement, MDD—medial disc displacement, LDD—lateral disc displacement, DDwR—disc displacement with reduction, DDwoR—disc displacement without reduction. * 2 patients had both MDD and LDD.

## Data Availability

Data are available from the corresponding author upon reasonable request.
